# Left ventricular apical thrombus after systemic thrombolysis with recombinant tissue plasminogen activator in a patient with acute ischemic stroke

**DOI:** 10.1186/1476-7120-3-14

**Published:** 2005-05-26

**Authors:** Florian Doepp, Wasiem Sanad, Stephan J Schreiber, Gert Baumann, Adrian C Borges

**Affiliations:** 1 Department of Neurology, University Hospital Charité, Berlin, Germany; 2 Department of Cardiology, University Hospital Charité, Berlin, Germany

**Keywords:** thrombolysis, hypercoagulability, ischemic stroke, cardiac thrombus

## Abstract

**Background:**

Thrombolysis with recombinant tissue plasminogen activator (rtPA) is an established treatment in acute stroke. To prevent rethrombosis after rtPA therapy, secondary anticoagulation with heparin is commonly performed. However, the recommended time-point and extent of heparin treatment vary and are not well investigated.

**Case presentation:**

We report a 61-year-old man who developed an acute global aphasia and right-sided hemiparesis. Cranial CT was normal and systemic thrombolytic therapy with tPA was started 120 minutes after symptom onset. Low-dose subcutaneous heparin treatment was initiated 24 hours later. Transthoracic echocardiography (TTE) 12 hours after admission showed slightly reduced left ventricular ejection fraction (LVEF) but was otherwise normal. 48 hours later the patient suddenly deteriorated with clinical signs of dyspnea and tachycardia. TTE revelead a large left ventricular apical thrombus as well as a reduction of LVEF to 20 %. Serial further TTE investigations demonstrated a complete resolution of the thrombus and normalisation of LVEF within two days.

**Conclusion:**

Our case demonstrates an intracardiac thrombus formation following rtPA treatment of acute stroke, probably caused by secondary hypercoagulability. Rethrombosis or new thrombus formation might be an underestimated complication of rtPA therapy and potentially explain cases of secondary stroke progression.

## Background

Recombinant tissue plasminogen activator (rtPA) is an approved treatment option for acute ischemic stroke within three hours of symptoms onset. It is well known that reperfusion of ischaemic brain tissue, when performed in a timely manner, improves clinical outcome [1,2]. However, there is evidence, that rtPA induces a secondary activation of coagulation cascades and inhibition of endogenous fibrinolysis [3,4]. These hemostatic changes might contribute to severe complications of rtPA treatment such as vessel reocclusion or recurrent stroke. Therefore, an adjuvant anticoagulation with heparin is performed to prevent reocclusion in myocardial infarction [5] and after intra-arterial thrombolysis of the basilar artery [6]. However, no studies or consensus exist regarding early anticoagulation after systemic thrombolysis in acute stroke.

We present a patient with acute ischaemic stroke who developed a transient left ventricular apical thrombus after systemic thrombolysis.

## Case presentation

A 61-year-old man was admitted to the Emergency Department immediately after he experienced global aphasia and right-sided hemiparesis. The clinical diagnosis of acute cerebral ischaemia in the middle cerebral artery (MCA) territory was made by a neurologist. A cranial CT-scan showed no early signs of cerebral infarct and no intracranial bleeding. Atrial fibrillation was recognised on the initial ECG and an cerebral infarct of cardiac embolic origin was assumed. The patient had no history of stroke but suffered from coronary heart disease and sustained an anterior cardiac wall infarction in the past. Further known vascular risk factors were hypertension and smoking. Even so, the patient took no medication at the time of admission.

Thrombolysis with 70 mg rt-PA (0.9 mg/kg) was started two hours after the onset of symptoms. The neurological status during and after rt-PA treatment remained unchanged. Transthoracic echocardiography (TTE) performed 12 hours after initiation of thrombolytic therapy revealed a moderately reduced left ventricular systolic function (ejection fraction: 41%), left ventricular regional dyssynergy, and no intracardiac thrombus.

The D-Dimer antigen plasma concentration as a marker of coagulation activation was raised 24 hours after rt-PA treatment and showed a tendency to normalisation during the following days.

Extracranial Duplexsonography revealed moderate arteriosclerotic plaque formation in the carotid bulb on both sides but no hemodynamic stenosis of the carotid and vertebral arteries. Using transcranial duplexsonography a severe stenosis was found in the proximal segment of the left middle cerebral artery. No diabetes or hyperlipidaemia were diagnosed. Cerebral MRI, twenty-four hours after lysis revealed a cortical infarct with a clinically asymptomatic haemorrhagic transformation in the left-sided MCA territory. Antithrombotic treatment with subcutaneous low-dose heparin (Dalteparin 2/d) was initiated 24 hours after thrombolysis. The fluid balance was continuously monitored and kept net 0 ml/24 hours to avoid fluid overload.

**Figure 1:**
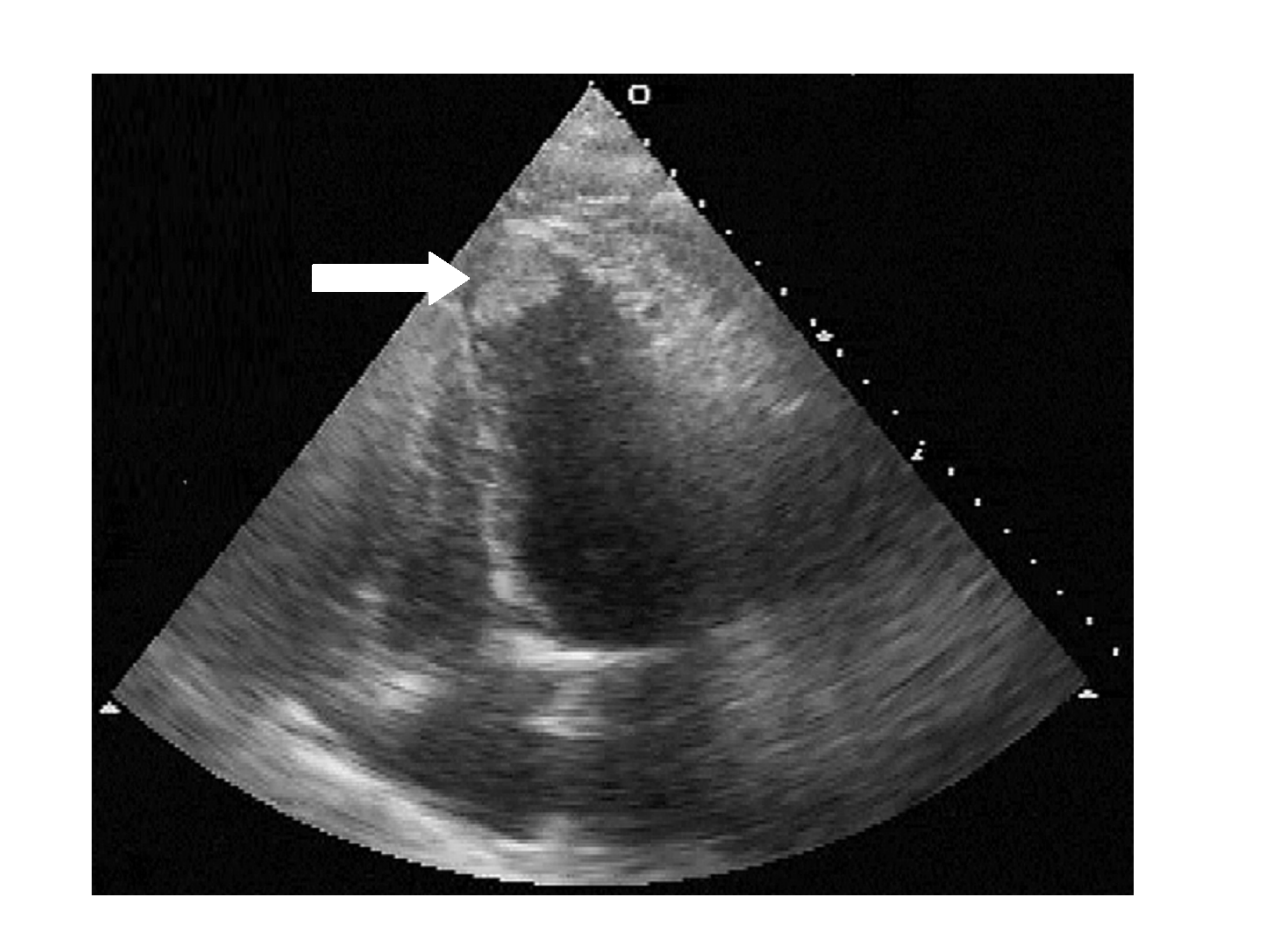
Cardiac duplex ultrasound image (4-chamber view), obtained 2 days after thrombolysis showing the thrombus attached to left ventricular apex (arrow).

On the 3^rd ^day after admission, the patient developed acute dyspnea, tachypnea, tachycardia, and an elevated blood pressure. Acute myocardial infarction was excluded on the basis of ECG and blood tests (CK, CK-MB, Troponin were normal). A CT-scan detected no pulmonary embolism, but massive bilateral pleural effusion. TTE revealed an apical thrombus measuring 2.0 × 2.0 cm in the left ventricle (figure 1) with left ventricular ejection fraction reduced to 20%. The thrombus, was fixed to the hypokinetic anteroseptal wall. Serial TTE performed two days later revealed complete resolution of the thrombus and hypokinesia; with a left ventricular ejection fraction of 40%. In the mean time the patient recovered from the acute cardiac decompensation. The neurological clinical picture remained stable during the acute phase with a slight improvement of aphasia and hemiparesis within the following 5 weeks.

## Discussion

Systemic rt-PA thrombolysis in acute stroke has been implemented into daily clinical practice during the last decade. The treatment within three hours of stroke appears to be effective in reducing the neurological deficit [1,7]. However, the use of rtPA therapy remains limited due to the narrow time-to-treatment windows and the potential complications of intracranial haemorrhage [7]. The optimal time-point of anticoagulation after systemic thrombolysis is unclear due to a lack of evidence. In accordance with the NINDS-protocol anticoagulation is interdicted within 24 hours after thrombolysis [1]. Also, latest guidelines for early stroke management recommend no antithrombotic therapy within 24 hours after application of rt-PA although there is increasing evidence for vessel reocclusion in about one third of the patients [8]. However, in clinical practice the use of anticoagulative agents seems to be much more heterogeneous [9].

Some authors have reported a significantly higher incidence of parenchymal haematomas if thrombolysis was immediately followed by intravenous or subcutaneous heparin administration [10]. However, recent analyses have shown that full-dose intravenous anticoagulant treatment within 24 hours does not increase the incidence of parenchymal hemorrhage [9].

rt-PA has a short biological half-life of 8–12 minutes but alters the physiological balance between coagulation and anticoagulation for a longer time period [11]. After discontinuation of rtPA infusion several mechanisms, potentially leading to a secondary hypercoagulability status have been discussed. For instance, the activation of plasminogen activator inhibitor-1, resulting in suppression of endogenous fibrinolysis [4], the increase of thrombin generation and activity [3], the increase of thrombin-antithrombin-III-complex levels [12] or the induction of hypoperfusion in ischaemic brain tissue [11]. The procoagulant response to rt-PA has been shown to persist up to 72 hours [3].

Otherwise, clinical studies of fibrinolytic therapy in myocardial infarction show, that early heparin treatment starting immediately after thrombolysis significantly decreases the risk of cardiac vessel reocclusion [5]. The capacity to attenuate the increased coagulation activity seems to be similar, regardless if low-molecular-weight or unfractionated heparin is used [13]. In stroke patients with an occlusion in the posterior cerebral circulation undergoing intra-arterial thrombolysis, heparin treatment is also known to reduce the rate of early reocclusion [6].

In our patient, treated with low dose heparin, an apical thrombus developed within 72 hours after thrombolytic therapy, strongly suggesting a causal relationship of secondary hypercoagulability and thrombus formation. The patient's history and risk profile suggests an increased risk for cardiac events [14]. We postulate, that he developed an acute coronary syndrome (dyspnea and tachycardia) on the basis of the pre-existing cardiac disease with kinetic disturbance which subsequently enabled the formation of a ventricular thrombus – promoted by the risk factors hypercoagulability, atrial fibrillation and previous myocardial infarction [15]. The transient increase of D-Dimer probably indicates the state of hypercoagulability after rt-PA treatment [3]. The low-dose heparin therapy in our patient was not sufficient to prevent thrombus development although a number of studies have shown that sufficient systemic anticoagulation with heparin is able to do so [16].

Coronary angiography was not performed, considering the cerebral state of the patient and the observation that repeated ECGs were normal and cardiac enzymes not elevated. The clinical status stabilised spontaneously and the cardiac thrombus resolved within two days without initiation of a specific therapy, further supporting the hypothesis of a temporary status of hypercoagulation after thrombolysis.

Another hypothesis for the temporary cardiac detoriation in our patient could be an acute neurogenic stunned myocardium. This phenomenon is described as sudden, reversible left ventricular dysfunction with abnormal left ventricular wall motion and reduced ejection fraction. Levels of creatine kinase MB and troponin may be elevated and ECG alterations as depression or elevation of the ST segment or T wave inversion can be observed. However, acute neurogenic myocardial stunning has so far only been reported after subarachnoid hemorrhage [17-20] and in isolated cases of subdural hematoma [21] and Guillain-Barré syndrome [22]. We found no evidence in the literature for acute neurogenic myocardial stunning after stroke. In addition, our patient suffered from coronary artery disease whereas neurogenic myocardial stunning has been reported in patients without cardiac disease. Finally, the cardiac failure in our patient occurred on the 3^rd ^day and not directy after stroke which makes the diagnosis of neurogenic stunned myocardium further unlikely [17,20,21].

In summary, our case demonstrates an intracardiac thrombus formation following rtPA treatment of acute stroke, probably caused by secondary hypercoagulability. Rethrombosis or new thrombus formation might be an underestimated complication of rtPA therapy and potentially explain cases of secondary stroke progression. Early systemic anticoagulation with heparin might reduce the risk of rethrombosis but also increase the risk of a bleeding complication. Systematic studies concerning the incidence of thrombus formation after rtPA therapy and the effects of different post-thrombolysis anticoagulation strategies are required to assess the clinical relevance of the discussed secondary hypercoagulability. Closed echocardiographic monitoring in stroke patients treated with systemic thrombolysis might be useful for early detection of the described potential cardiac complications especially because repeated measurement of ECG and cardiac enzymes alone might fail.
